# Supplementary material for: Nonspecific Amyloid Aggregation of Chicken Smooth-Muscle Titin: In Vitro Investigations
Source: Int J Mol Sci. 2023 Jan 5;24(2):1056. doi: 10.3390/ijms24021056 (PMC9861715; doi:10.3390/ijms24021056)
Supplement: Supplementary file 1 [file ijms-24-01056-s001.zip › +Supplementary File S2.pdf]

Data on the amyloidogenic segments predicted for 6 sequences using the FoldAmyloid software (amino acids are numbered starting from 1 for each sequence)

>sp|A6BM71|**Ig-like_1** (6-98 A6BM71)
PTFTQPLQSVVALEGSAATFEAHISGFPVPEVSWYRDGQVLSAATLPGVQISFSDGRAKL
VIPSVTEANSGRYTIQATNGSGQATSTAELLVT
Amyloidogenic regions found 2
59 - 63 (length 5)
89 - 93 (length 5)

>sp|A6BM71|**Ig-like_10** (1805-1896 A6BM71)
PKIFERIQSQTVAQGTDAHFRVRVVGKPDPECQWFRNGVQIERTDRIYWYWPEDNVCEL
VIRDVTADDSASIMVKAVNIAGETSSHAFLLVQ
Amyloidogenic regions found 5
19 - 24 (length 6)
33 - 37 (length 5)
46 - 51 (length 6)
57 - 63 (length 7)
87 - 92 (length 6)

>sp|A6BM71|**Ig-like_23** (2966-3056 A6BM71)
PQIIQELQPTTVESGKPARFCAIISGKPQPKVSWYKDDQQLSPGFKCKFLHDAQEYTLLLI
ETFPEDSAVYTCEAKNDYGVATTSASLSVE
Amyloidogenic regions found 3
19 - 23 (length 5)
47 - 51 (length 5)
56 - 63 (length 8)
>sp|A6BM71|**Fibro_2** (7428-7520 A6BM71)
PGPPQNVEVTDVNRFGRTLTWEAPEYDGGSPITGYVIELRNRASIKWEPTMTTGADELS
AVLTDVVENEEYFFRVRAQNMVGVGKPSHPTRAV
Amyloidogenic regions found 2
34 - 39 (length 6)
71 - 75 (length 5)

>sp|A6BM71|**Fibro_3** (7529-7622 A6BM71)
PSGNINLDHSDQTKTSVQLTWEPPLEDGGSPILGYIIERKEEGTDKWIRCNPKLVPACAFK
VTGLKAGSSYYYRVSAENAAGVSDPAEAIGPLT
Amyloidogenic regions found 2
33 - 38 (length 6)
71 - 75 (length 5)

>sp|A6BM71|**Fibro_5** (7825 - 7913 A6BM71)
PDPPENVKWRNPTSKGIFLTWEPPKYDGGARIKGYLVEKCQRGTDKWEICGEPVIETKM
EVTKLKEGEWYAYRVKALNRIGASKPSKPT
Amyloidogenic regions found 3
17 - 21 (length 5)
33 - 38 (length 6)
69 - 75 (length 7)
As an example, we calculated the content of amyloidogenic segments in random titin domains (three Ig-like and three FnIII-like). From the data obtained, there were at least two amyloidogenic segments in each of the calculated domains and, e.g., the Ig10 domain had five such segments.

This is yet another proof of the high predisposition of this protein to form amyloid aggregates in the case of domain unfolding.
