# Supplementary material for: Nonspecific Amyloid Aggregation of Chicken Smooth-Muscle Titin: In Vitro Investigations
Source: Int J Mol Sci. 2023 Jan 5;24(2):1056. doi: 10.3390/ijms24021056 (PMC9861715; doi:10.3390/ijms24021056)

Molecular weight and content of titin isoforms in isolated protein preparations

<u>Gel 1</u> Two titin bands, **1102** kDa and **1629** kDa **37.8%/62.2%** (lower/upper)

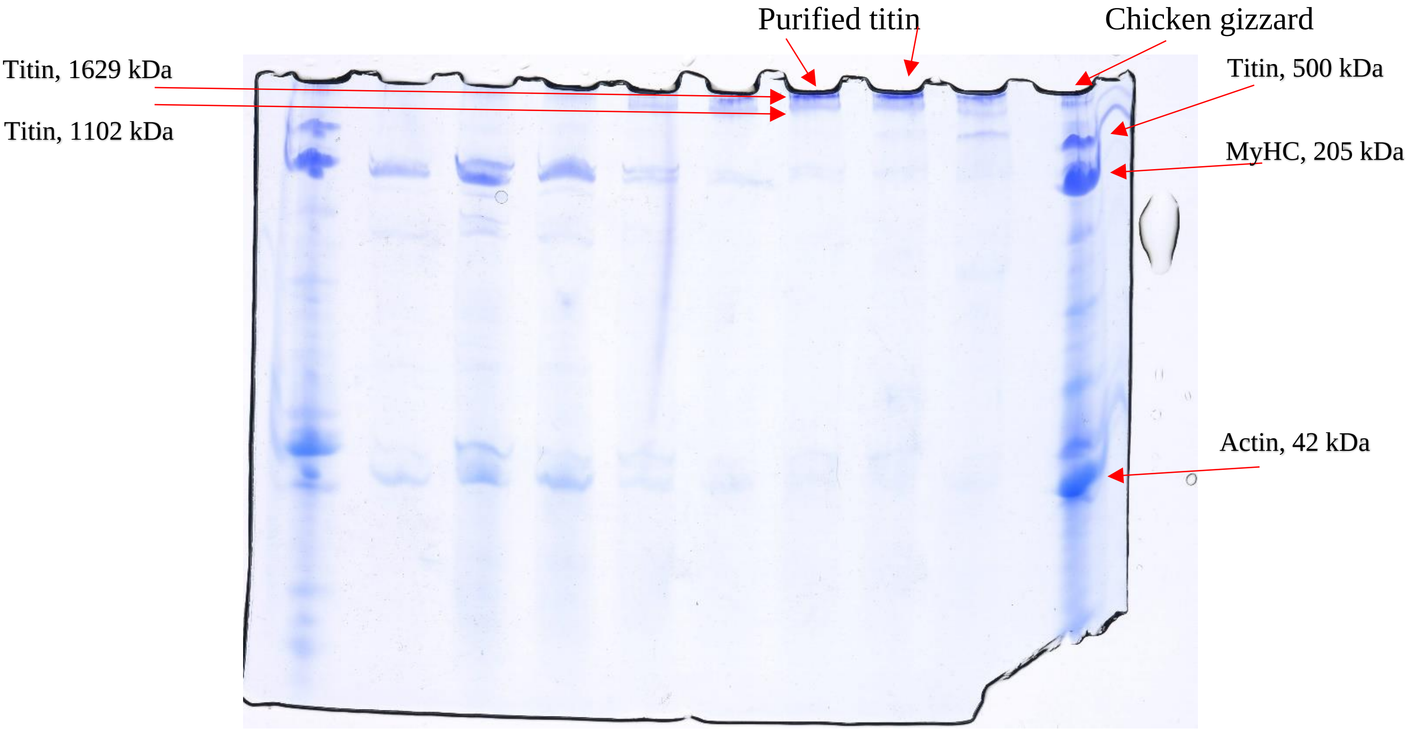

Gel 2 Two titin bands, **1061** kDa and **1322** kDa **25% / 75%** (lower / upper)

1115 kDa and **1357** kDa

1127 kDa and **1399** kDa

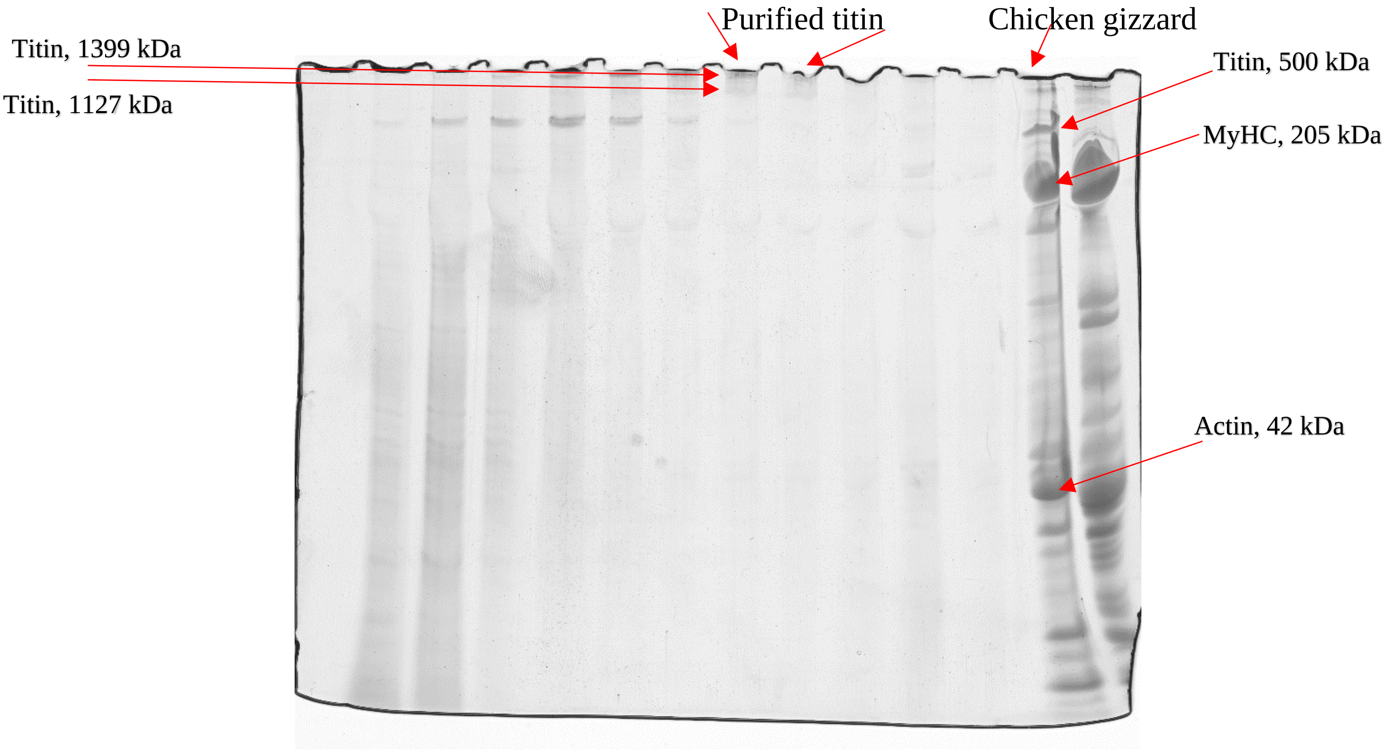

Gel 3 Two bands, **1625** kDa and **1954** kDa **31.4% / 68.6%** (lower / upper)

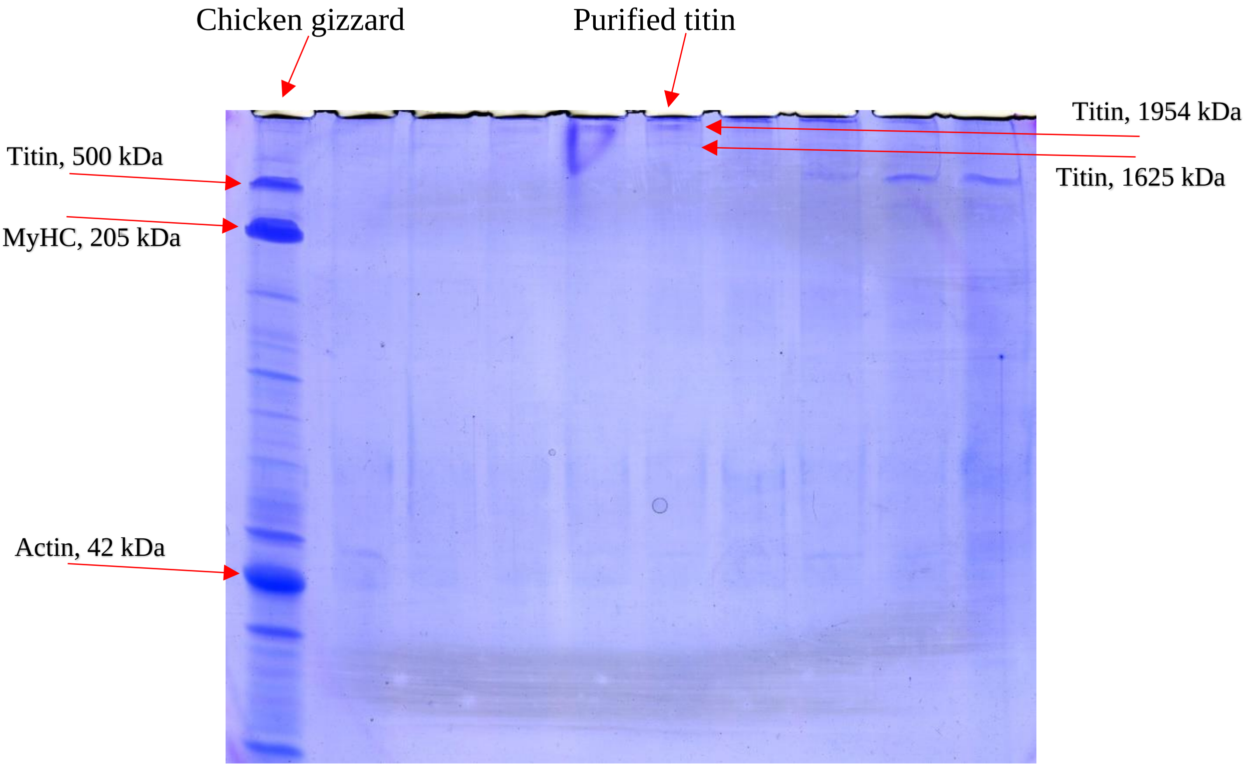

Gel 4		
	Two bands, **1345** kDa and **1819** kDa	**22.4% / 77.6%** (lower/upper)
	**1354** kDa and **1819** kDa	**33.7% / 66.3%** (lower/upper)
	**1233** kDa and **1785** kDa	**38.4% / 61.6%** (lower/upper)

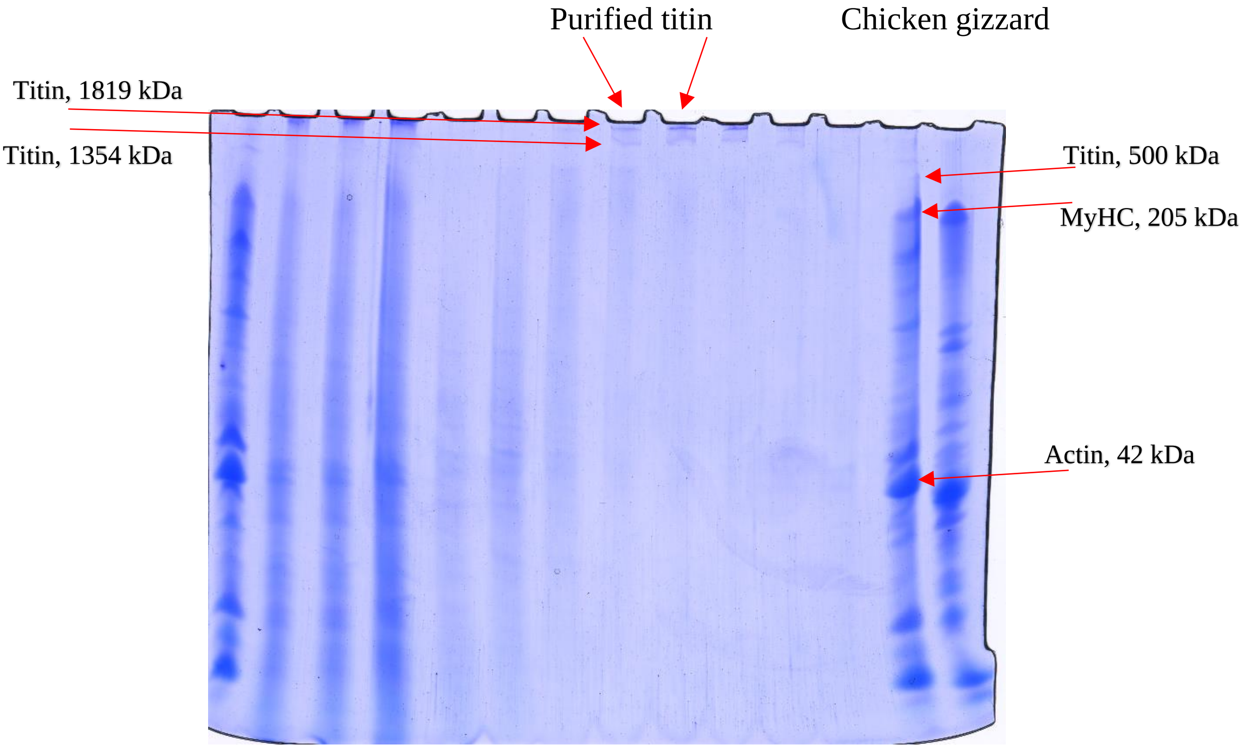

Thus, the preparations of isolated titin contained two isoforms or, possibly, an isoform and a proteolytic fragment with the molecular weights of **1635±245 kDa** (upper band, n = 8) and **1245±189 kDa** (lower band, n = 8).

The contents of a lower-molecular-weight and higher-molecular-weight titin in the preparations were **31.5% / 68.5%** (n = 6).

Examples of molecular weight calculation

1

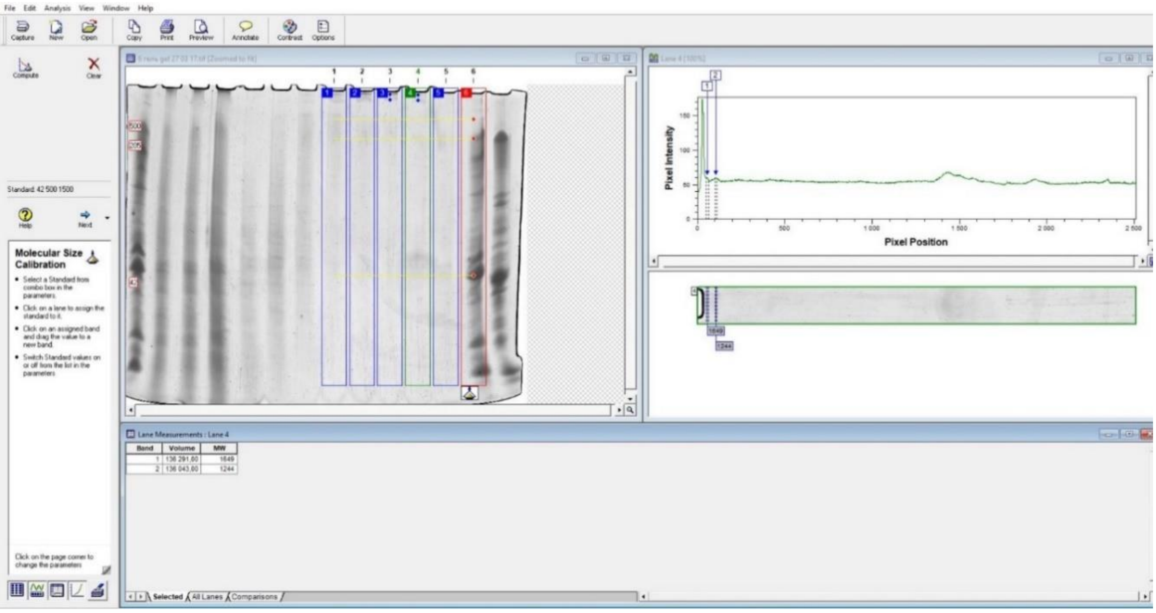

2

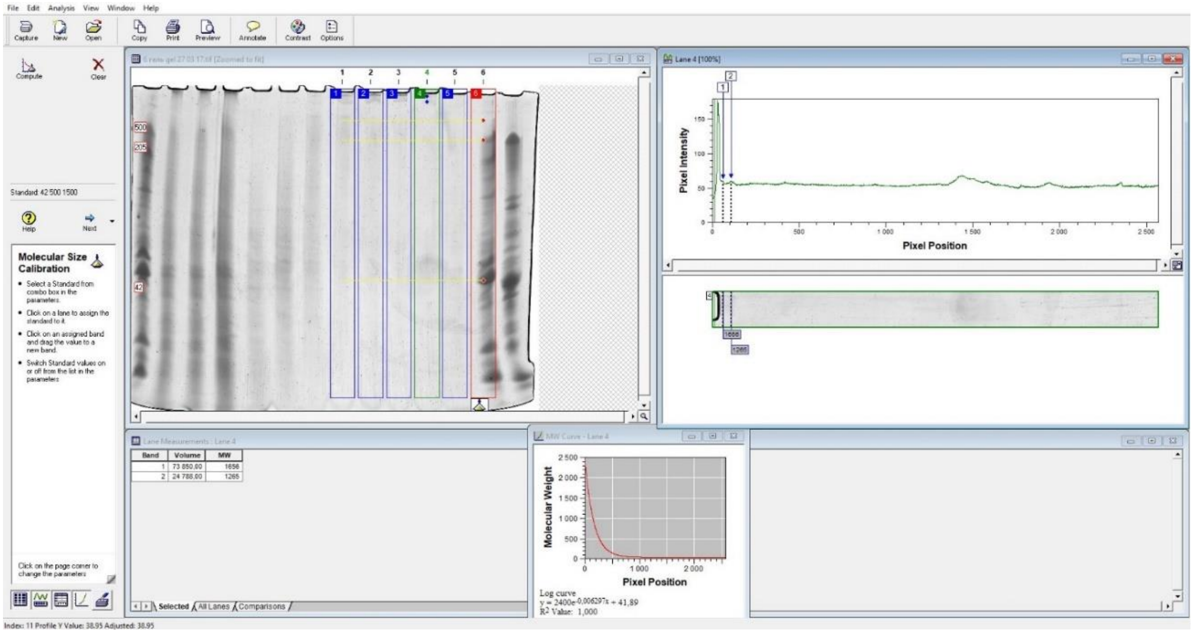

3

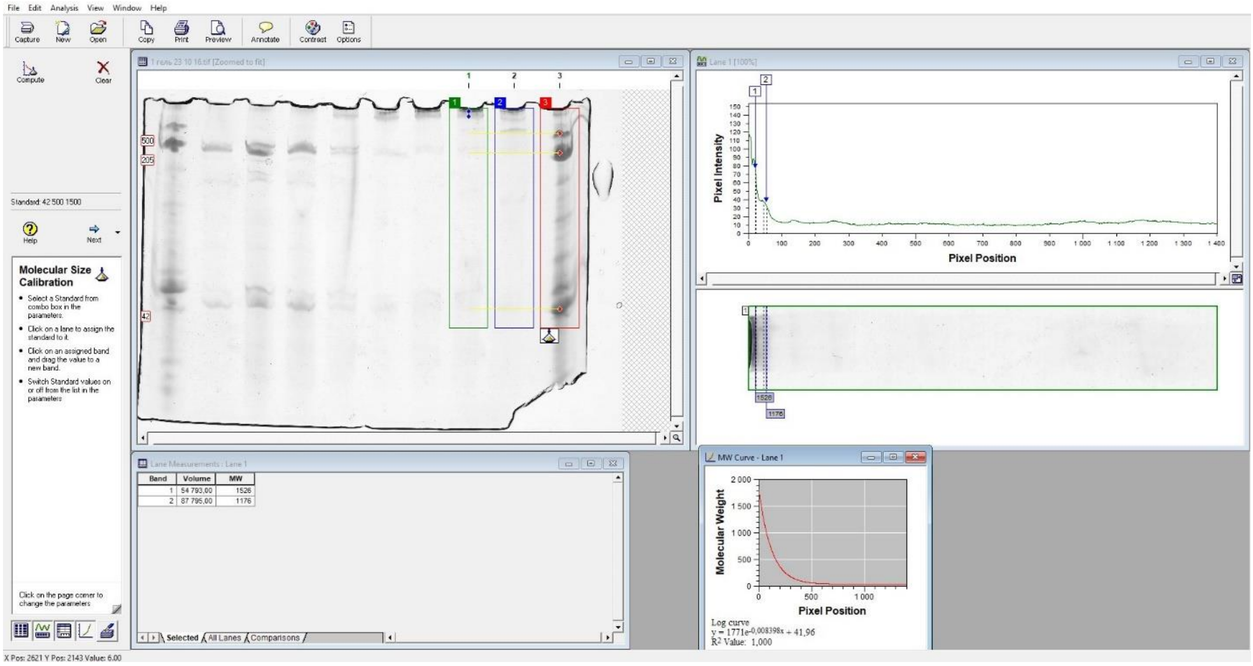

Supplement: Supplementary file 1 [file ijms-24-01056-s001.zip › +Supplementary File S3.pdf]
