# Supplementary figures and images for: Nonspecific Amyloid Aggregation of Chicken Smooth-Muscle Titin: In Vitro Investigations
Source: Int J Mol Sci. 2023 Jan 5;24(2):1056. doi: 10.3390/ijms24021056 (PMC9861715; doi:10.3390/ijms24021056)

AFM images of a buffer solution (0.15 M glycine–KOH, pH 7.0–7.5) containing no titin

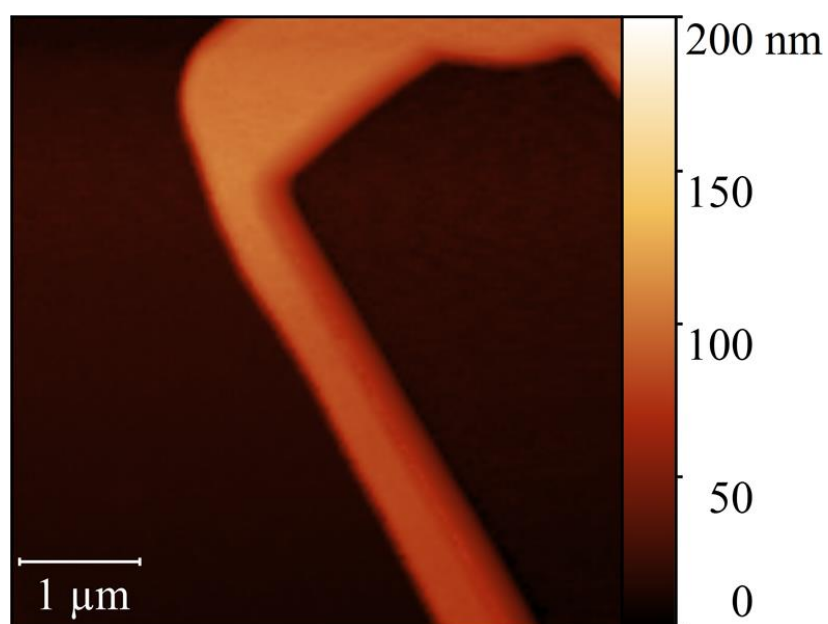

a

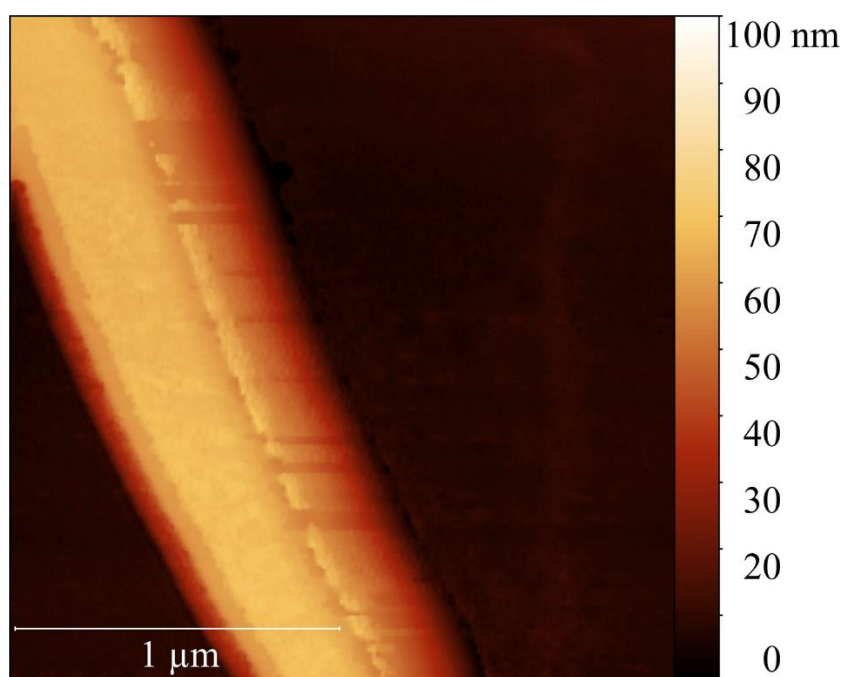

b

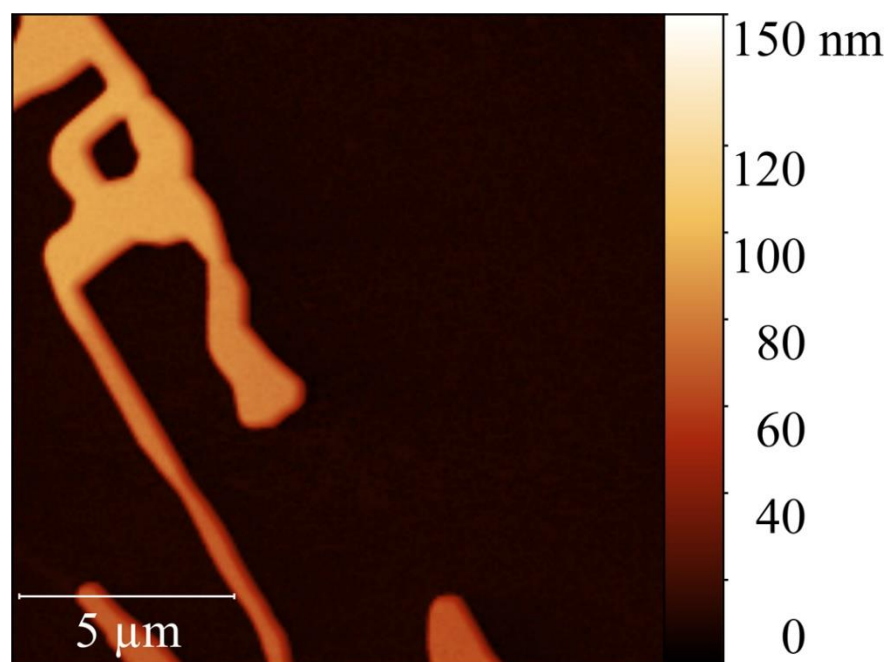

c

Supplement: Supplementary file 1 [file ijms-24-01056-s001.zip › +Supplementary File S4.pdf]
